# Parametric estimation of cross-frequency coupling

**DOI:** 10.1016/j.jneumeth.2015.01.032

**Published:** 2015-03-30

**Authors:** B.C.M. van Wijk, A. Jha, W. Penny, V. Litvak

**Affiliations:** a Wellcome Trust Centre for Neuroimaging, UCL Institute of Neurology, 12 Queen Square, WC1N 3BG London, UK; b Sobell Department of Motor Neuroscience and Movement Disorders, UCL Institute of Neurology, 33 Queen Square House, Queen Square, WC1N 3BG London, UK

**Keywords:** Nested oscillations, Beta band, Deep brain stimulation (DBS), Magnetoencephalography (MEG), Power fluctuations, Connectivity

## Abstract

**Background:**

Growing experimental evidence suggests an important role for cross-frequency coupling in neural processing, in particular for phase-amplitude coupling (PAC). Although the details of methods to detect PAC may vary, a common procedure to estimate the significance level is the comparison of observed values to those of at least 100 surrogate time series. When scanning large parts of the frequency spectrum and multiple recording sites, this could amount to very large computation times.

**New method:**

We demonstrate that the general linear model (GLM) allows for a parametric estimation of significant PAC. Continuous recordings are split into epochs, of a few seconds duration, on which an *F*-test can be performed. We compared its performance against traditional non-parametric permutation tests in both simulated and experimental data.

**Results:**

Our method was able to reproduce findings of phase-amplitude coupling in local field potential recordings obtained from the subthalamic nucleus in patients with Parkinson's disease. We also show that PAC may be detected between the subthalamic nucleus and cortical motor areas.

**Comparison with existing method(s):**

Although the GLM slightly underestimated significance compared to permutation tests in the simulations, for experimental data the two methods produced highly similar results. Computation times were drastically lower for the GLM. Furthermore, we demonstrate that the GLM can be easily extended by including additional predictors such as low-frequency amplitude to test for amplitude-amplitude coupling.

**Conclusions:**

The GLM forms an adequate and computationally efficient approach for detecting cross-frequency coupling with the flexibility to add other explanatory variables of interest.

## Highlights

• We revisit the general linear model (GLM) approach to cross-frequency coupling.
• Continuous time series were split into epochs for parametric statistical tests.
• The GLM and permutation tests produced similar results in experimental data.
• The GLM offers a good trade-off between computation time and statistical power.
• Other predictors such as amplitude-amplitude coupling can be easily included.

## 1 Introduction

Electrophysiological signals are traditionally partitioned into different frequency bands that show characteristic modulations with cognition and behaviour (Buzsáki, 2006). The functional roles of these frequency bands are usually studied in isolation by looking at the spectral power of signals originating from single brain regions or by applying linear measures of functional connectivity like coherence or phase-locking between signals of separate regions. Although this has revealed important insights into brain functioning, it is likely that neural processing also relies on the interaction *between* frequency bands (Canolty and Knight, 2010). In fact, activity at different frequencies could be coupled in several ways involving either the phase, amplitude or the frequency of the signals (Jirsa and Muller, 2013). This opens up an additional range of mechanisms through which information processing might be achieved. Indeed, growing empirical evidence suggests that forms of cross-frequency coupling are present in recorded brain signals and may show modulations with task performance or pathology.

To date, phase-amplitude coupling (PAC) has been the best studied form of cross-frequency coupling, which arises when the phase of a low frequency oscillation modulates the amplitude of a high frequency oscillation. A seminal example is the theta-gamma coupling in rodent hippocampal activity (Tort et al., 2008, 2009), also frequently observed in humans across a range of frequencies and brain regions (e.g., Canolty et al., 2006; Maris et al., 2011; Miller et al., 2012). Some findings suggest a functional role for PAC, for example the pronounced theta-gamma PAC in hippocampus during periods of decision making (Tort et al., 2008) and the increase of PAC with the number of items kept in working memory (Axmacher et al., 2010). Moreover, PAC may even serve as a mechanism to encode the order of items in memory (Lisman and Jensen, 2013). Remarkably, an increased level of PAC could also be indicative of pathology, as was found for beta-gamma PAC in the motor cortex of patients with Parkinson's disease (de Hemptinne et al., 2013). Studying aspects of PAC might therefore be helpful for understanding both normal and abnormal neural activity patterns.

Methods to detect PAC differ between studies but are usually either based on the entropy of the phase-amplitude distribution (Tort et al., 2008) or the computation of the resultant vector length of amplitude values projected on the phase circle (Canolty et al., 2006). The latter could also be cast in the form of a general linear model (GLM) (Penny et al., 2008; Özkurt and Schnitzler, 2011b). Several recent studies suggested possible improvements of these methods aimed at increasing the detectability of significant coupling. These include the selection of high amplitude time frequency bins (Dvorak and Fenton, 2014), tracking of instantaneous frequencies (van Zaen et al., 2013; Pittman-Polletta et al., 2014), and accounting for non-sinusoidal wave forms of the phase frequency (Kramer and Eden, 2013).

Regardless of the details of PAC detection algorithms, most methods depend on surrogate data to determine the significance of observed findings. Typically, for each permutation the PAC is recalculated with either the phase or amplitude signal shifted in time, and the measured PAC values are compared to a distribution of at least 100 surrogate values. As often one is not fully certain of the precise amplitude and phase frequencies at which effects of interest occur, PAC is calculated for a whole range of frequency combinations. This may result in large computation times when surrogate PAC values have to be estimated for each of these combinations, particularly when investigating inter-regional coupling on sensor or source level as the number of channel combinations could become large. In this paper we show that parametric statistical analysis could be applied when PAC is estimated using a GLM, hence obviating the need for computing surrogate data. Another advantage of the GLM is that *p*-values could be adequately corrected for multiple comparisons using random field theory (Brett et al., 2003; Kilner et al., 2005), which takes into account the dependencies between PAC estimates at adjacent frequencies.

Although phase-amplitude coupling has been the most reported form of cross-frequency coupling, it does not exclude the potential occurrence of other forms of coupling. In particular, the slow fluctuations in amplitude of low and high frequency bands may be coupled via amplitude-amplitude correlations (Bruns and Eckhorn, 2004; de Lange et al., 2008). We demonstrate that the GLM framework could be easily extended to include amplitude-amplitude (AAC) coupling in addition to phase-amplitude coupling. This allows for disentangling the contribution of phase and amplitude components to cross-frequency coupling, as well as testing for possible co-occurrence of both forms. First we will illustrate the validity of our approach using simulations of known ratios of phase-amplitude and amplitude-amplitude coupling. We subsequently apply our method to invasive recordings of the subthalamic nucleus (STN) obtained from deep brain stimulation electrodes in Parkinson's disease patients. Using simultaneously recorded magnetoencephalography (MEG), we also look for cross-frequency coupling between the STN and cortical activity.

## 2 Methods

### 2.1 GLM

**Fig. 1:**
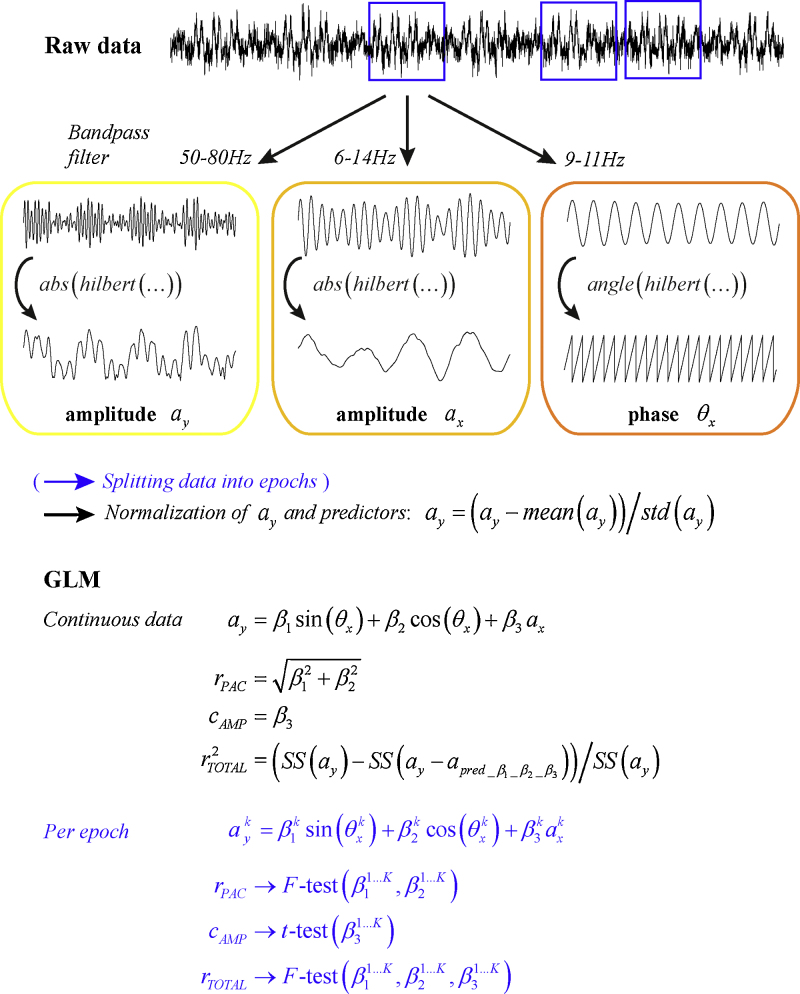
Schematic overview of the processing steps required to apply the GLM. First of all, the instantaneous amplitude and phase for the intended frequencies need to be extracted using bandpass filtering and Hilbert transformation. *Z*-transformation of these time series ensures that PAC and AAC estimates are normalised and comparable to other existing measures. The *β*-coefficients of the GLM are estimated in two ways: (1) using the entire data length available; (2) by first dividing the continuous data into shorter epochs and re-estimating the *β*-coefficients for each epoch. The latter estimates are subsequently used to assess the significance of the overall coupling values in the indicated way.

The GLM for phase-amplitude coupling was introduced by Penny et al. (2008). We here extend this framework to include amplitude-amplitude coupling and evaluate its ability to detect significant frequency combinations both in simulations and real data. The procedure for this is summarised in Fig. 1. The amplitude of high-frequency signal components acts as the data to be explained by the GLM, while the predictors are formed by the phase and amplitude of low-frequency signal components. These can be obtained by following the conventional approach of bandpass filtering and the use of Hilbert transformation to extract the instantaneous phase via *θ*_*x*_ = mod(*angle*(*hilbert*(*x*)), 2*π*), and amplitude via *a*_*y*_ = *abs*(*hilbert*(*y*)). Here *x* and *y* denote the low and high-frequency signal components respectively that have been obtained after bandpass filtering either the same or two different time series. Note that the bandwidth of the filters should be adjusted according to which feature is extracted. It is imperative that the bandwidth of the filter for the amplitude is wide enough to include the centre frequency ± phase frequency in order to extract an amplitude signal that could fluctuate with the same frequency as the phase (Berman et al., 2012; Dvorak and Fenton, 2014). This means that when testing for phase-amplitude coupling between 10 Hz and 65 Hz, the bandpass filter should at least be [55 75] Hz to be able to detect such a coupling. On the other hand, the bandwidth for extracting the phase of the signal should be narrow as to obtain an accurate phase estimate of the centre frequency (Chavez et al., 2006). We, therefore, applied two different filters for the lower frequency, the second one with a slightly broader bandwidth to extract the slowly fluctuating amplitude of the low-frequency signal.

The GLM is constructed as:

(1) $$a_{y}=\beta_{1}\sin(\theta_{x})+\beta_{2}\cos(\theta_{x})+\beta_{3}a_{x}+\varepsilon \text{,}$$

and can be estimated using least squares. To ensure that estimation of the *β*-coefficients leads to normalised measures of phase-amplitude and amplitude-amplitude coupling, the dependent variable *a*_*y*_ and the predictors are transformed to have zero mean and unit variance: *a*_*y*_ = (*a*_*y*_ − mean(*a*_*y*_))/std(*a*_*y*_). The same applies to sin(*θ*_*x*_), cos(*θ*_*x*_), and *a*_*x*_. In this case, the amount of explained variance in amplitude that can be explained by the phase equals $r_{PAC}^{2}=(SS(a_{y})-SS(a_{y}-a_{pred\_\beta_{1}\_\beta_{2}}))/SS(a_{y})$, for which $r_{PAC}=\sqrt{\beta_{1}^{2}+\beta_{2}^{2}}$. The latter is equivalent to the more commonly used resultant vector length approach of $\left|a_{y}e^{i\theta_{x}}\right|$ after normalisation (see also Özkurt and Schnitzler, 2011b). It is bounded by the interval [0 1]. Amplitude-amplitude coupling is represented by the third coefficient *c*_*AMP*_ = *β*_3_. Like a correlation coefficient, its values can range from -1 to 1. Finally, the total amount of explained variance by including both the low-frequency phase and amplitude in the model is given by $r_{TOTAL}^{2}=(SS(a_{y})-SS(a_{y}-a_{pred\_\beta_{1}\_\beta_{2}\_\beta_{3}}))/SS(a_{y})$ Normalisation of the amplitude values eliminates the need for a constant term in the GLM to capture the mean amplitude.

An overall estimate of cross-frequency coupling is obtained by including all recorded time points in the GLM. However, the following approach can be used to assess the significance of the estimated values. Instead of estimating the GLM on continuous data, the time-series are first divided into *K* epochs of shorter duration. Each of these epochs *k* enters a separate GLM for which the *β*-coefficients are estimated. This yields a whole set of *β*_1_, *β*_2_ and *β*_3_ values. The consistency of *β*-coefficients over epochs can subsequently be evaluated with a parametric statistical test. For *r*_*PAC*_ and *r*_*TOTAL*_ an *F*-test can be used on $\left[\beta_{1}^{1\ldots K},\beta_{2}^{1\ldots K}\right]$, and $\left[\beta_{1}^{1\ldots K},\beta_{2}^{1\ldots K},\beta_{3}^{1\ldots K}\right]$ respectively. For *c*_*AMP*_ a *t*-test can be performed on $\beta_{3}^{1\ldots K}$. The frequency combinations with a *p*-value below the chosen significance level indicate which cross-frequency estimates obtained from the continuous data are significant. In contrast to *r*_*PAC*_, the estimates for *β*_1_ and *β*_2_ will be centred around zero across epochs in case of no significant PAC. This is a unique feature of our GLM approach, allowing to directly test against the null hypothesis of no significant coupling. For the alternative approaches of vector length and entropy one would have to resort to surrogate data for comparison.

All analyses were performed in Matlab and the code is made freely available for general use through the SPM toolbox (http://www.fil.ion.ucl.ac.uk/spm; Litvak et al., 2011b) via the function called *spm_eeg_cfc.m*. The GLM for cross-frequency coupling between EEG/MEG source level and a reference channel is implemented in the SPM toolbox for *Data Analysis in Source Space (DAiSS)* (http://code.google.com/p/spm-beamforming-toolbox).

## 3 Simulations

We first demonstrate the validity of our GLM approach using simulated time series with constructed PAC and AAC under different levels of noise. The contribution of PAC and AAC to the signal was systemically varied to test whether and when the GLM was able to correctly estimate them. To generate these time series we used

(2) $$\begin{array}{l} x_{amp}=\sin(F_{amp\_low}2\pi t)\\ x_{phase}=\sin(F_{phase}2\pi t+\theta_{0x})\\ x=(A_{0}+x_{amp})x_{phase}\\ y=(A_{0}+w_{1}x_{phase}+w_{2}x_{amp})\sin(F_{amp}2\pi t+\theta_{0y})\\ z_{signal}=x+y\\ z_{noise}=\rho \sigma (z_{signal})\xi (t)\\ z_{full}=z_{signal}+z_{noise} \end{array}$$

The signals are essentially a mix of a low-frequency oscillator and a high-frequency oscillator, where the amplitude of the high-frequency oscillator is modulated by both the phase and fluctuations in amplitude of the low-frequency oscillator. To mimic the frequencies observed in our examples of real data, we chose *F*_*amp*_ = 205 Hz for the high-frequency and *F*_*phase*_ = 18.033 Hz for the low-frequency oscillator. The slow fluctuations in amplitude of the low-frequency oscillator were set at $F_{amp\_low}=1.95\;\text{Hz}$. Non-integer frequencies were chosen to avoid exact numbers of cycles per epoch that would make the shuffling of epochs for permutation tests ineffective (see below). Amplitude fluctuations occurred around a baseline level of *A*_0_ = 3. The contributions of the phase and amplitude of the low-frequency oscillator to the high-frequency amplitude were independently weighted by $w_{1}$ and $w_{2}$, respectively, and varied from [0:0.25:2]. On top of the true signal we added white observation noise ξ(t) that was scaled by the factor *ρ* with values [0:0.25:1, 2:10]. For each parameter setting 20 realizations were performed with independent initial phases *θ*_0_. The GLM was fitted with amplitude and phase signals extracted from bandpass filters targeted around the true coupling frequencies. The bandwidth chosen for the low-frequency amplitude was ±4 Hz, for phase ±2 Hz and for amplitude ±26 Hz.

We explored to what extent the sampling rate, number of epochs and epoch length could influence the estimated overall coupling values and the associated statistical inference. For this we compared sampling rates of [600 1200] Hz, epoch lengths of [2 4] s and [15 30] for the number of epochs. These numbers were chosen to leave the total number of samples constant between selected comparisons. For example, in this way the effect of doubling the number of epochs could be directly compared to the effect of doubling the length of the epochs. The total number of samples per generated time series was either 18,000, 36,000, 72,000, or 144,000. As the number of false positives detected by the GLM turned out to be independent of these settings, we focused on the hit rates of identifying true coupling under different levels of noise. The performance for *r*_*PAC*_ and *c*_*AMP*_ was compared to that of the standard permutations approach for which we created 100 surrogate time series by shuffling the high-frequency amplitude across epochs within the continuous time series. We performed this analysis twice, with $w_{1}=1,w_{2}=0$ (hence only PAC present), and $w_{1}=0,w_{2}=1$ (only AAC present). For both cases, we increased the number of realizations to 200 for a better estimation of the hit rates.

## 4 Real data

The ability of the GLM method to capture known cross-frequency coupling in real data sets is crucial for its utility. We here capitalize on the findings of previous studies (Özkurt et al., 2011a; López-Azcárate et al., 2010) who showed a clear phase-amplitude coupling between the beta band (∼20 Hz) and frequencies around 300 Hz in local field potential recordings from the subthalamic nucleus (STN) in patients with Parkinson's disease. Using our own data set with similar recordings (Litvak et al., 2011a) we show a number of cases where significant cross-frequency coupling could be detected using the GLM. Furthermore, as MEG recordings were simultaneously obtained, we show an example of PAC between the STN and ipsilateral cortical motor areas.

Full details of the experimental recordings can be found in Litvak et al. (2011a). In brief, bilateral STN local field potential (LFP) activity was recorded at the same time as 275 channel MEG (CTF/VSM MedTech, Vancouver, Canada) a few days after patients underwent STN electrode implantation for deep brain stimulation treatment. LFPs were recorded using four intracranial STN electrodes on each side and off-line converted to a bi-polar montage between adjacent contacts. Sampling rate was set at 2400 Hz. Patients were instructed to keep still during the recordings and to focus their eye gaze on a fixation cross without performing any explicit task. Recordings took place in separate sessions while the patient was either “on” or “off” dopaminergic medication. We here present a subset of the total dataset for illustrative purposes. Shown examples are taken from four different patients and selected STN electrodes (patient 1: on medication – left STN; patient 2: off medication – right STN; patient 3: on medication – right STN; patient 4: off medication – left STN).

The continuous recordings of about 3 min were divided into shorter epochs of 3.4 s. Those epochs containing spike artefacts were discarded, which resulted in a range of 14–52 epochs analysed per example. After bandpass-filtering, the first and last 167 ms of each epoch were removed to avoid filter ringing. GLMs were estimated for centre frequencies between 5 and 26 Hz with 1 Hz steps for the low-frequency component and between 100 and 400 Hz with 2 Hz steps for the high-frequency component. Bandwidth for obtaining the phase signals was ±1 Hz, for the amplitude fluctuations of the low-frequency component ±4 Hz, and for the high-frequency amplitude signals ±26 Hz. All epochs were concatenated for estimating the overall cross-frequency coupling, while the single epochs each entered a separate GLM to test for significance at *α* = .01 or *α* = .05.

We compared the significance of *r*_*PAC*_ values obtained from the GLM to those obtained by the conventional non-parametric approach of shuffling either phase or amplitude in time. This was done by permuting the order of epochs for the amplitude signals and recalculating *r*_*PAC*_. In this way, 200 surrogate time series were computed and *r*_*PAC*_ values higher than the .95 percentile of the permuted values were taken to be significant. As a third way to test for significance, we made use of the threshold that was analytically derived by Özkurt (2012). Given the large number of statistical tests due to the various combinations of low and high centre frequencies, we also used a Bonferroni correction and controlled the family wise error-rate using random field theory (Brett et al., 2003). The latter takes into account the correlation of neighbouring frequencies and is therefore less conservative. In addition, we computed the expected number of false positives under the GLM method when applied to 100 surrogate time series generated as described above.

Finally, we computed PAC between high-frequency amplitude (bandpass filter 300 ± 100 Hz) of the STN and beta band phase (22 ± 7 Hz) of the simultaneously recorded MEG data. The MEG signals were first projected to source space using linearly constrained minimum variance beamforming (see Litvak et al., 2010). For this we used a single shell head model based on an individual structural MRI scan that was transformed to template space. The resolution of the source space was set to 10 mm × 10 mm × 10 mm. Beamformer filters were calculated after band-pass filtering the data with [15 29] Hz in order to obtain optimal estimates of beta band source activity. Regularisation was set at 0.01%. Source data were extracted by multiplying the channel data with the estimated beamformer filter weights per grid point (resulting in ‘virtual electrodes’). The Hilbert phase of these time series subsequently entered the GLM as predictor, while the high-frequency amplitude of the STN again served as the independent variable. The GLM was estimated for all grid points and significance was determined in the same way as described for within STN coupling.

## 5 Results

### 5.1 Simulations

**Fig. 2:**
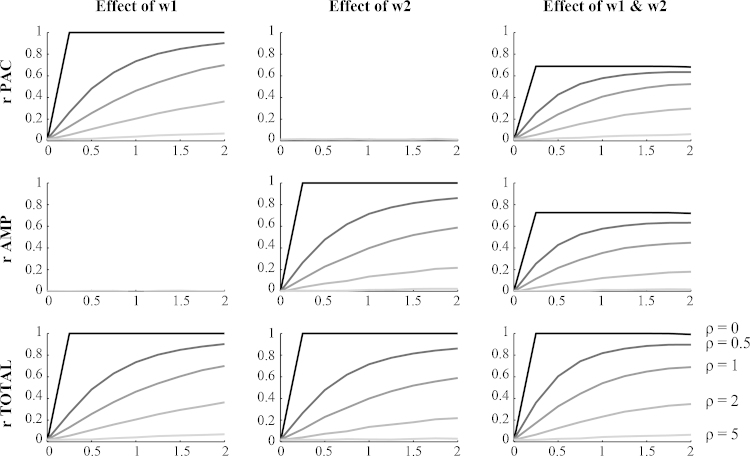
Estimated cross-frequency coupling under different settings of PAC and AAC. Sampling rate was fixed at 600 Hz, epoch length at 2 s and number of epochs at 15. Different lines represent different quantities *ρ* of noise added, hence different signal-to-noise ratios. In the left column the amount of AAC was set at 0, while the amount of PAC varied. Vice versa for the second column. The third column shows the estimates obtained for simulations where *w*_1_ equalled *w*_2_.

Fig. 2 shows the estimated overall cross-frequency coupling obtained from simulated data with known proportions of PAC and AAC. *r*_*PAC*_ and *r*_*AMP*_ only deviated from zero when there was real coupling present in the signals. Without the presence of noise, the coupling is correctly detected at a maximum value of 1, which gradually declines with lower signal-to-noise levels. This effect could be counterbalanced by having stronger coupling coefficients. This is the case because a stronger coupling does not alter the standard deviation of *z*_*signal*_ much, therefore the signal-to-noise ratio was minimally affected while more amplitude coupling was preserved in the Hilbert transformed signals. In case both PAC and AAC are present in the signal, neither $r_{PAC}^{2}$ or $r_{AMP}^{2}$ was able to explain all the variance. The combined measure of *r*_*TOTAL*_ could then be used to judge the total strength of cross-frequency coupling.

**Fig. 3:**
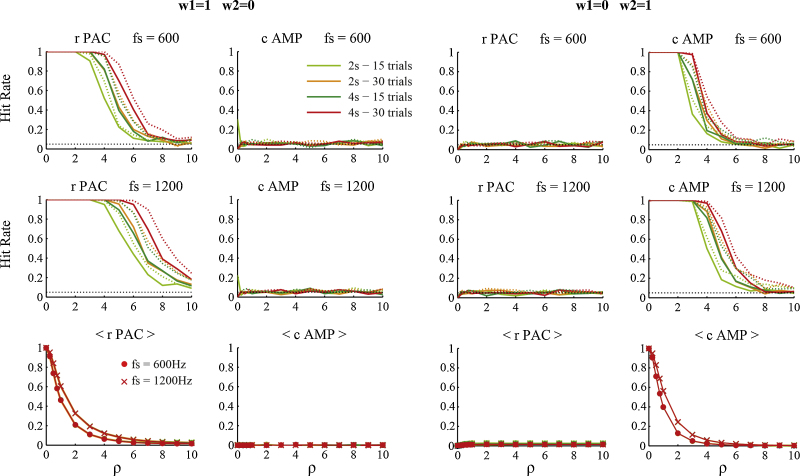
The effect of varying sampling rate, epoch length and number of epochs on the statistical inference and the estimated coupling values. The panels on the left show results for the presence of PAC only, the right panels for the presence of AAC only. The proportion of correctly assigned coupling estimates as significant is designated by the hit rate. The total amount of data available influenced the hit rate, not the division of epoch length versus number of epochs. In addition, higher sampling rate led to more correctly identified significant values, as well as higher overall estimates. The latter were little affected by the total number and length of epochs used. Solid lines designate the results for the GLM, dotted lines for the conventional permutation tests, which had slightly higher hit rates. All plots are a function of *ρ* – the relative amount of observation noise. Significance level was set at *α* = .05.

The performance of the GLM in assigning significance to estimated coupling values is shown in Fig. 3. Significant coupling was more often detected when more data samples in total were available. For these simulations, the hit rates for 15 epochs of 4 s and 30 epochs of 2 s were equal. This means that the exact division of the total signal into epochs is not very important. Hit rates also increased with higher sampling rate. Compared to conventional permutation tests the GLM detected significant coupling less often, and therefore has lower statistical power. This might be due to the fact that the Hilbert amplitude of Gaussian noise inherently follows a chi distribution with 2 degrees of freedom. The performance of the GLM might therefore be more conservative as the residuals are not strictly normally distributed under high levels of observation noise. For low SNRs both methods become equally ineffective in detecting cross-frequency coupling. False positive rates were around 5% for the cases where no PAC or AAC was present in the signals, both for the GLM and the permutation tests. Remarkably, the sampling rate also had a larger effect on the overall coupling estimates than the amount of data available, for which the curves were indistinguishable. Hence the number of samples per cycle appears to be more important than the duration of the recordings.

## 6 Real data

**Fig. 4:**
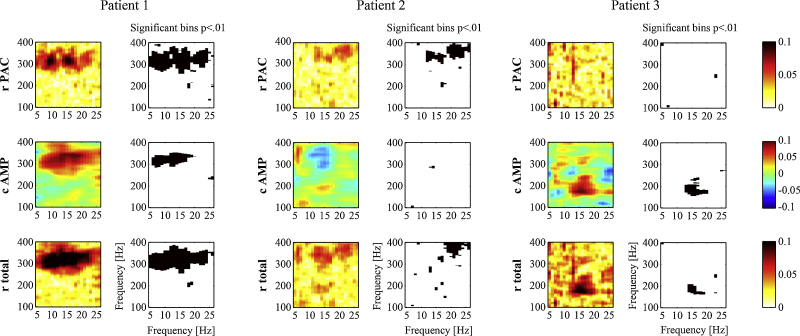
Results of applying the GLM to data recorded from the subthalamic nucleus in three different Parkinson's disease patients. Frequency bins indicated in black were found to be significant at *p* < .01. There is a clear inter-subject variability regarding the presence of significant PAC and AAC. Either of these may occur separately but also simultaneously at the same or different frequencies. The three patients were selected from our data set to demonstrate this diversity.

To demonstrate the applicability of the GLM to real data, we analysed invasive recordings from deep brain stimulation electrodes in the STN of patients with Parkinson's disease. We first analysed the STN-LFP data in isolation looking for evidence of “local” PAC within the STN. In line with previous studies (Özkurt et al., 2011a; López-Azcárate et al., 2010), we were able to detect significant PAC between the phase of beta oscillations and the amplitude of frequencies around 300 Hz. We present examples of three patients in Fig. 4. These cases illustrate some of the heterogeneity that may occur in cross-frequency coupling between individuals. Whereas the first example shows both significant PAC and AAC across a wide range of alpha/beta and high frequencies, the other two examples only show either significant PAC or AAC in isolation. The first example shows that PAC may be accompanied by AAC at the same frequencies, which suggests a non-exclusive role for phase in controlling high-frequency amplitude. The total amount of variance explained by the full GLM containing both cross-frequency measures is given by $r_{TOTAL}^{2}$.

**Fig. 5:**
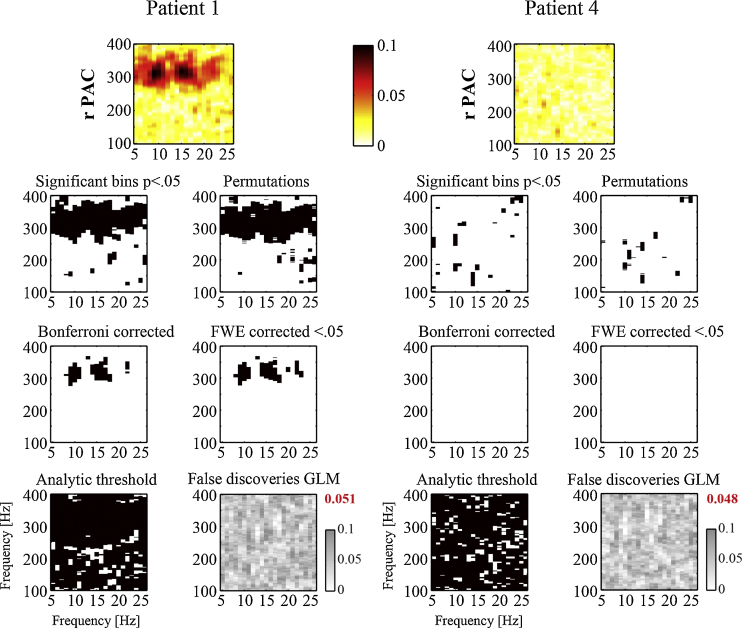
Illustration of the performance of different approaches of determining significance levels for PAC. The left example shows a case for which clear PAC seems to be present (patient 1). In the second case, no apparent PAC could be observed by eye (patient 4). The results of our GLM method are depicted in the top left panel. As can be seen, they bear a strong resemblance to the results obtained from conventional permutation tests (200 epoch-shuffled surrogate time series). In case clear significant results are found at uncorrected *p*-values, a correction for multiple comparisons could help pointing out the peak frequencies more precisely. Remarkably, the analytic threshold based on the theoretically expected distribution of normally distributed amplitudes failed to provide accurate results. Both in the case with and without clear PAC it assigned significance to most of the frequency combinations. Lastly, the GLM identified on average the expected 5% false positives at *α* = .05 when it was estimated for 100 surrogate time series (average percentage indicated in red). (For interpretation of the references to colour in this legend, the reader is referred to the web version of the article.)

The performance of different significance tests is shown in Fig. 5. Two examples are shown, one with apparent PAC clearly present and one without. There is a strong resemblance between the results of the GLM and the permutation tests. Apart from a few scattered points at mostly random frequency combinations, the pattern of significant values is highly similar. Out of the 3322 bins of frequency combinations in the spectrum, the GLM assigned significance to 122 bins (3.7%) that were not detected with the permutation tests for example 1, and 104 (3.1%) for example 2. Vice versa, the permutation tests assigned significance to 163 bins (4.9%) that were not detected with the GLM method for example 1, and 54 bins (1.6%) for example 2. These numbers are representative across our entire data set (140 spectra in total), for which on average 2.6% more significant bins were detected with the GLM versus 3.8% for the permutation tests. In rare cases, only the permutation tests assigned significance to PAC that seemed to be of a more transient nature, hence not consistently present across all epochs. Overall, the high similarity between results of the GLM and permutation tests across spectra supports the use of the GLM as an alternative to non-parametric testing.

By contrast, the analytically derived significance threshold (Özkurt, 2012) resulted in high numbers of significant values, both for the example with apparent PAC present as well as the one without. A correction for multiple comparisons brought out the frequencies for which the peak PAC occurred in the spectrum more clearly. The family wise error-rate correction performed close to the Bonferroni correction for these data. Finally as expected, the average percentage of false discoveries detected by the GLM in surrogate data was 5%. These were uniformly distributed across the spectrum and hence did not seem to bias results in a particular frequency range.

**Fig. 6:**
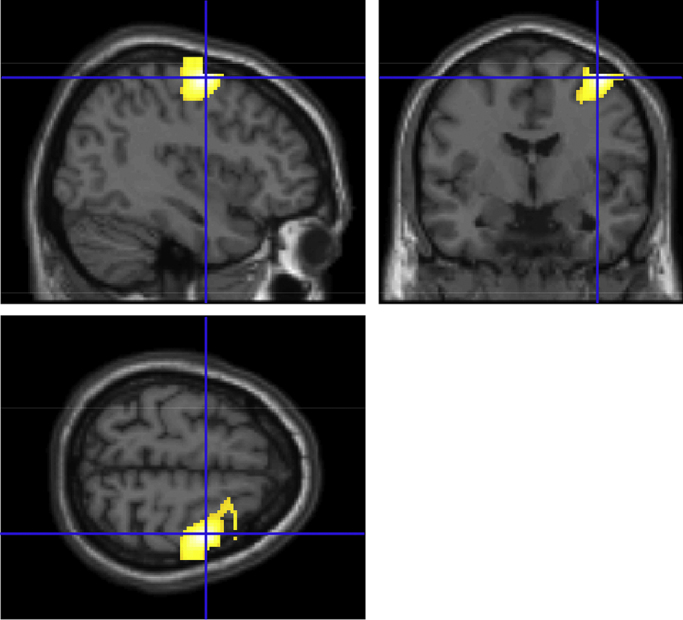
Detection of significant PAC between high-frequency amplitude of the STN and beta phase in cortical motor areas. Shown are the results of patient 2 from whom we analysed recordings from the right STN and simultaneously recorded MEG during rest. The MEG signals were projected to source space using beamforming. The GLM was estimated for all voxels and significance was determined in the same way as described for within STN coupling. As the combination of frequencies that gives rise to the highest PAC is unknown a priori, we selected broadband bandpass filters for the beta phase (22 ± 7 Hz) and the high-frequency amplitude (300 ± 100 Hz). Shown results are significant at *p* < .001. The indicated cluster is located in the right pre-motor area and has its peak value at MNI coordinates [46 −16 64]. The cluster remains significant after a whole brain FWE cluster-level correction of *p* = .01.

We then proceeded to look for PAC between the STN and cortical sources using both the STN-LFP and MEG data. High-frequency amplitude was again obtained from the STN recordings but now the beta-band phase from the source-projected MEG data was used as predictor. In this way, inter-regional cross-frequency coupling may be revealed. Although such coupling might be weaker and harder to detect, we did observe some examples for which significant coupling could be observed with ipsilateral motor areas. Fig. 6 shows an example of a patient for whom significant PAC could be detected between the left STN and the left the motor cortex.

## 7 Discussion

We have shown the applicability of parametric estimation of phase-amplitude and amplitude-amplitude coupling using a general linear model. Although its statistical power was slightly lower compared to permutation tests in the simulations, a comparison between the two for real data revealed a highly consistent pattern. The presented GLM allows for disentangling the contributions of PAC and AAC and detecting for which frequencies they occur simultaneously. The implementation is relatively simple and reduces computational cost by obviating the need for permutation tests. Moreover, the framework could be easily extended by including additional predictors that could be of interest to specific studies, for example to study other forms of coupling, to account for non-linearities in phases (non-sinusoidal waveforms) (Kramer and Eden, 2013) or to remove confounding factors. Cross-frequency terms might even be included in convolution models that explain time-frequency responses to presented stimuli (Litvak et al., 2013).

We were able to reproduce previously reported PAC in our own data set. Although the exact frequencies of PAC and AAC varied, significant values were found in a large number of patients, of which we show a few representative examples. In addition, we present a novel finding showing that the high-frequency amplitude is not only coupled to the beta phase within the STN, but a significant locking to the beta phase of cortical motor areas may also be detected. While it is known that these regions show beta band coherence (Litvak et al., 2011a), our findings might reflect a possible indirect path between cortical beta phase and high-amplitude activity in STN. In principle, the GLM also offers the possibility to include the phase or amplitude of other frequency bands or brain regions as additional regressors, to investiage whether the estimated cross-frequency coupling has explanatory value on top of the contribution of other regressors. A more in-depth investigation of cross-frequency coupling between STN and cortical regions will be conducted in a future study.

As demonstrated in the simulations, the quality of the estimated *β*-coefficients strongly depends on the signal-to-noise ratio, the amount of actual coupling present in the signal, and may improve with higher sampling rates. Likewise, the successful detection of significant values also depended on these settings. With more data points available to compute the Hilbert amplitude and phase, we postulate that higher sampling rates make the identification of PAC and AAC more accurate. Higher sampling rates even outweighed the effect of adding more data when it comes to estimating the overall coupling values. On the other hand, hit rates may be improved by including more and/or longer epochs in cases with relatively large observation noise. As we also observed empirically in our data set, the trade-off between epoch length and number of epochs is not very critical in detecting significant values. Obviously, the choice of epoch length is bounded by the fact that adequate numbers of epochs should remain in order to justify the *F*- or *t*-tests. Conversely, enough data samples should be available per epoch to estimate the *β*-coefficients.

Notably, the analytical significance threshold (Özkurt, 2012) led to large numbers of significant values for our data, even when no obvious PAC was present by eye. A closer look at the distribution of amplitude values revealed a right-skewed distribution, which violates the assumption of normally distributed amplitudes used in the derivation. Despite the vast reduction in computation time, it produced an unacceptably large number of false positives in our case. The GLM seems to offer a better compromise despite the somewhat lower statistical power compared to permutation tests. Although the computational cost may still be extensive as the GLM needs to be computed for individual epochs, it is substantially faster than computing *r*_*PAC*_ for large numbers of surrogate data. To give an example, the computation of the GLM for the data presented in Fig. 5a took 6 min and 40 s on our desktop computer (64-bit Windows 7, 3.20 GHz Intel Xeon CPU, 12 GB RAM). On the other hand, a permutation test of 200 surrogates of the same data took almost 159 min to compute (via the resultant vector length method). This represents a ∼24-fold reduction in computation time for the GLM.

After a Fisher *z*-transformation of the amplitude and GLM predictors, *r*_*PAC*_ becomes equivalent to the resultant vector length after correct normalisation. This standardisation ensures that *r*_*PAC*_ values could be interpreted quantitively, as the average amplitude level and the size of the fluctations have been made irrelvant. This also allows for comparisons between conditions that show differences in spectral power, although increases in signal-to-noise ratio might lead to better estimation of phase and amplitude and therefore to larger PAC values. When both PAC and AAC are present in the data, neither $r_{PAC}^{2}$ or $r_{AMP}^{2}$ will reach to 1, as shown in the simulations. It could be useful in those cases to look at the combined measure of $r_{TOTAL}^{2}$ instead.

The examples presented in this study are taken from individual subjects. It would however be relatively straightforward to perform a group analysis using GLM. The statistics are similar to that used for single epochs: an *F*-test for [*β*_1_, *β*_2_] to test for *r*_*PAC*_ and [*β*_1_, *β*_2_, *β*_3_] for *r*_*TOTAL*_, and a *t*-test for *c*_*PAC*_. Instead of the *β*-coefficients for single epochs, the *β*-coefficients obtained from the GLMs applied to the continuous data are entered. Another contrast that could be tested is that of the full GLM compared to a reduced version containing only phase or amplitude predictors. In this way, one could explicitly test for the presence of added AAC to PAC or vice versa.

Detection of cross-frequency coupling in multi-channel recordings could be improved by using an optimal linear combination of sensors that maximizes the cross-frequency estimates, as was shown for multi-frequency phase-locking (Nikulin et al., 2012) and amplitude-amplitude correlations (Dähne et al., 2014). Related to this is the method by Sampson et al. (2012), where phase-amplitude coupling is modelled parametrically with a Fourier series and a spatial filter is sought to maximize the coupling strength. Besides giving a best possible estimate of the coupling strength, these methods also help localising where the coupling occurs (on sensor/channel level).

It should be noted that various factors could give rise to false detections of cross-frequency coupling. A clear and in-depth discussion on this topic is presented by Aru et al. (2014). They describe how non-linearities of the low-frequency oscillation could readily lead to detectable PAC with higher frequencies. Also the simultaneous modulation of phase and amplitude by an external factor could give rise to apparent PAC even though no mechanistic interaction between the two exists. This may especially play a role in event-related designs where an external stimulus may modulate both the low-frequency phase and high-frequency amplitude independently. Finally, care should be taken that the bandwidths of the low and high frequencies do not overlap to avoid spurious coupling. This is why it is difficult to look at phase-amplitude coupling between neighbouring frequency bands such as alpha and beta. It is less of a concern when the phase and amplitude are extracted from different signals coming from separate brain regions. Taking these issues into account, detected cross-frequency coupling might not always have high biological relevance despite statistical tests indicating its significance.

As with most measures of functional connectivity, the PAC and AAC estimated by the GLM are merely correlational. They do not reveal the neurophysiological mechanisms that are causing the cross-frequency coupling. The low-frequency phase might prescribe when the high-frequency amplitude is high or low but likewise, it could be rhythmic fluctuations in amplitude that drive lower-frequency activity. Invasive recordings are required to see whether these oscillations are generated by separate types of neurons and may, therefore, provide a means of neural information processing. Importantly, Spaak et al. (2012) identified that cross-frequency coupling could occur between neurons in different cortical layers. Using layer-specific recordings from primate V1, they revealed a robust coupling between the alpha phase of infragranular layers and the gamma amplitude of granular and supragranular layers. In addition, they found evidence for a negative amplitude-amplitude coupling by showing that gamma amplitude tended to be high when alpha amplitude was low. These findings hint at a mechanistic role of cross-frequency coupling within the cortical column, and could possibly extend to inter-regional interactions.

Another way of investigating the generative mechanisms behind cross-frequency coupling is through computational modelling. In particular biologically detailed models such as the canonical microcircuit model proposed by Bastos et al. (2012) may prove useful in interpreting experimental findings as they have done in dynamic causal modelling (Moran et al., 2013), especially because they describe the dynamics of neural subgroups within different layers and their interactions. The GLM method proposed in this paper may provide a step towards this by providing a reliable method to detect patterns of cross-frequency coupling in data recordings.

## References

[bib0190] Aru J., Aru J., Priesemann V., Wibral M., Lana L., Pipa G. (2014). Untangling cross-frequency coupling in neuroscience. Curr Opin Neurobiol.

[bib0010] Axmacher N., Henseler M.M., Jensen O., Weinreich I., Elger C.E., Fell J. (2010). Cross-frequency coupling supports multi-item working memory in the human hippocampus. Proc Natl Acad Sci U S A.

[bib0015] Bastos A.M., Usrey W.M., Adams R.A., Mangun G.R., Fries P., Friston K.J. (2012). Canonical microcircuits for predictive coding. Neuron.

[bib0020] Berman J.I., McDaniel J., Liu S., Cornew L., Gaetz W., Roberts T.P. (2012). Variable bandwidth filtering for improved sensitivity of cross-frequency coupling metrics. Brain Connect.

[bib0025] Brett M., Penny W., Kiebel S. (2003). An introduction to random field theory. Human brain function II.

[bib0030] Bruns A., Eckhorn R. (2004). Task-related coupling from high- to low-frequency signals among visual cortical areas in human subdural recordings. Int J Psychophysiol.

[bib0035] Buzsáki G. (2006). Rhythms of the brain.

[bib0040] Canolty R.T., Edwards E., Dalal S.S., Soltani M., Nagarajan S.S., Kirsch H.E. (2006). High gamma power is phase-locked to theta oscillations in human neocortex. Science.

[bib0045] Canolty R.T., Knight R.T. (2010). The functional role of cross-frequency coupling. Trends Cogn Sci.

[bib0050] Chavez M., Besserve M., Adam C., Martinerie J. (2006). Towards a proper estimation of phase synchronization from time series. J Neurosci Methods.

[bib0055] Dähne S., Nikulin V.V., Ramírez D., Schreier P.J., Muller K.R., Haufe S. (2014). Finding brain oscillations with power dependencies in neuroimaging data. Neuroimage.

[bib0060] de Hemptinne C., Ryapolova-Webb E.S., Air E.L., Garcia P.A., Miller K.J., Ojemann J.G. (2013). Exaggerated phase-amplitude coupling in the primary motor cortex in Parkinson disease. Proc Natl Acad Sci U S A.

[bib0065] de Lange F.P., Jensen O., Bauer M., Toni I. (2008). Interactions between posterior gamma and frontal alpha/beta oscillations during imagined actions. Front Hum Neurosci.

[bib0070] Dvorak D., Fenton A.A. (2014). Toward a proper estimation of phase–amplitude coupling in neural oscillations. J Neurosci Methods.

[bib0075] Jirsa V., Müller V. (2013). Cross-frequency coupling in real and virtual brain networks. Front Comput Neurosci.

[bib0080] Kilner J.M., Kiebel S.J., Friston K.J. (2005). Applications of random field theory to electrophysiology. Neurosci Lett.

[bib0085] Kramer M.A., Eden U.T. (2013). Assessment of cross-frequency coupling with confidence using generalized linear models. J Neurosci Methods.

[bib0090] Lisman J.E., Jensen O. (2013). The theta–gamma neural code. Neuron.

[bib0095] Litvak V., Eusebio A., Jha A., Oostenveld R., Barnes G.R., Penny W.D. (2010). Optimized beamforming for simultaneous MEG and intracranial local field potential recordings in deep brain stimulation patients. Neuroimage.

[bib0100] Litvak V., Jha A., Eusebio A., Oostenveld R., Foltynie T., Limousin P. (2011). Resting oscillatory cortico-subthalamic connectivity in patients with Parkinson's disease. Brain.

[bib0105] Litvak V., Jha A., Flandin G., Friston K. (2013). Convolution models for induced electromagnetic responses. Neuroimage.

[bib0195] Litvak V., Mattout J., Kiebel S., Phillips C., Henson R., Kilner J. (2011). EEG and MEG data analysis in SPM8. Comput Intell Neurosci.

[bib0115] López-Azcárate J, Tainta M, Rodríguez-Oroz MC, Valencia M, González R, Guridi J, et al. Coupling between beta and high-frequency activity in the human subthalamic nucleus may be a pathophysiological mechanism in Parkinson's disease. J Neurosci 2010;30:6667–77. 10.1523/JNEUROSCI.5459-09.2010 PMC6632566 20463229

[bib0120] Maris E., van Vugt M., Kahana M. (2011). Spatially distributed patterns of oscillatory coupling between high-frequency amplitudes and low-frequency phases in human iEEG. Neuroimage.

[bib0125] Miller K.J., Hermes D., Honey C.J., Hebb A.O., Ramsey N.F., Knight R.T. (2012). Human motor cortical activity is selectively phase-entrained on underlying rhythms. PLoS Comput Biol.

[bib0130] Moran R., Pinotsis D.A., Friston K. (2013). Neural masses and fields in dynamic causal modeling. Front Comput Neurosci.

[bib0135] Nikulin V.V., Nolte G., Curio G. (2012). Cross-frequency decomposition: a novel technique for studying interactions between neuronal oscillations with different frequencies. Clin Neurophysiol.

[bib0140] Özkurt T.E. (2012). Statistically reliable and fast direct estimation of phase-amplitude cross-frequency coupling. IEEE Trans Biomed Eng.

[bib0145] Özkurt T.E., Butz M., Homburger M., Elben S., Vesper J., Wojtecki L. (2011). High frequency oscillations in the subthalamic nucleus: a neurophysiological marker of the motor state in Parkinson's disease. Exp Neurol.

[bib0150] Özkurt T.E., Schnitzler A. (2011). A critical note on the definition of phase–amplitude cross-frequency coupling. J Neurosci Methods.

[bib0155] Penny W.D., Duzel E., Miller K.J., Ojemann J.G. (2008). Testing for nested oscillation. J Neurosci Methods.

[bib0160] Pittman-Polletta B., Hsieh W.H., Kaur S., Lo M.T., Hu K. (2014). Detecting phase–amplitude coupling with high frequency resolution using adaptive decompositions. J Neurosci Methods.

[bib0165] Sampson A.L., Babadi B., Prerau M.J., Mukamel E.A., Brown E.N., Purdon P.L. (2012). Beamforming approach to phase–amplitude modulation analysis of multi-channel EEG. Conf Proc IEEE Eng Med Biol Soc.

[bib0170] Spaak E., Bonnefond M., Maier A., Leopold D.A., Jensen O. (2012). Layer-specific entrainment of gamma-band neural activity by the alpha rhythm in monkey visual cortex. Curr Biol.

[bib0175] Tort A.B., Komorowski R.W., Manns J.R., Kopell N.J., Eichenbaum H. (2009). Theta–gamma coupling increases during the learning of item-context associations. Proc Natl Acad Sci U S A.

[bib0180] Tort A.B., Kramer M.A., Thorn C., Gibson D.J., Kubota Y., Graybiel A.M. (2008). Dynamic cross-frequency couplings of local field potential oscillations in rat striatum and hippocampus during performance of a T-maze task. Proc Natl Acad Sci U S A.

[bib0185] van Zaen J., Murray M.M., Meuli R.A., Vesin J.M. (2013). Adaptive filtering methods for identifying cross-frequency couplings in human EEG. PLOS ONE.

